# TGF-β1 Increases GDNF Production by Upregulating the Expression of GDNF and Furin in Human Granulosa-Lutein Cells

**DOI:** 10.3390/cells9010185

**Published:** 2020-01-10

**Authors:** Jingwen Yin, Hsun-Ming Chang, Yuyin Yi, Yuanqing Yao, Peter C.K. Leung

**Affiliations:** 1 School of Medicine, Nankai University, Tianjin 300071, China; christina_yin@outlook.com; 2 Department of Obstetrics and Gynaecology, University of British Columbia, and BC Children’s Hospital Research Institute, Vancouver, BC V5Z 4H4, Canada; changobs@hotmail.com (H.-M.C.); yuyinyi0805@gmail.com (Y.Y.)

**Keywords:** GDNF, FURIN, TGF-β1, human granulosa cells, SMAD signaling

## Abstract

Glial cell line-derived neurotrophic factor (GDNF) is expressed at a high level in the human ovary and GDNF signaling is involved in the direct control of follicular activation and oocyte maturation. Transforming growth factor-β1 (TGF-β1) plays an important role in the regulation of various ovarian functions. Furin is an intracellular serine endopeptidase of the subtilisin family that is closely associated with the activation of multiple protein precursors. Despite the important roles of GDNF and TGF-β1 in the regulation of follicular development, whether TGF-β is able to regulate the expression and production of GDNF in human granulosa cells remains to be determined. The aim of this study was to investigate the effect of TGF-β1 on the production of GDNF and its underlying mechanisms in human granulosa-lutein (hGL) cells. We used two types of hGL cells (primary hGL cells and an established immortalized hGL cell line, SVOG cells) as study models. Our results show that TGF-β1 significantly induced the expression of GDNF and furin, which, in turn, increased the production of mature GDNF. Using a dual inhibition approach combining RNA interference and kinase inhibitors against cell signaling components, we showed that the TβRII type II receptor and ALK5 type I receptor are the principal receptors that mediated TGF-β1-induced cellular activity in hGL cells. Additionally, Sma- and Mad-related protein (SMAD)3 and SMAD4 are the downstream signaling transducers that mediate the biological response induced by TGF-β1. Furthermore, furin is the main proprotein convertase that induces the production of GDNF. These findings provide additional regulatory mechanisms by which an intrafollicular factor influences the production of another growth factor through a paracrine or autocrine interaction in hGL cells.

## 1. Introduction

Initially purified and cloned in 1993, glia cell line-derived neurotrophic factor (GDNF) is characterized as a critical trophic factor that promotes morphological differentiation and survival of midbrain dopaminergic neurons [1]. Subsequent research has demonstrated that GDNF is a potent survival factor for central motoneurons and may have a therapeutic potential in treating certain neurodegenerative diseases [2,3]. Outside the central nervous system, GDNF is also expressed in various human tissues, including the urogenital, digestive and reproductive systems [4,5,6,7]. In particular, the mRNA transcripts and mature proteins of GDNF and functional receptors of GDNF (GFRα1 and RET) are expressed at high levels in the principal cells (granulosa cells and oocytes) of the ovaries and testes in humans [6,7]. Recent evidence obtained from animal experiments and clinical information suggests a putative role for the GDNF signaling pathway in the regulation of multiple ovarian functions, including primordial follicle activation, follicular growth and oocyte maturation [8]. Specifically, animal studies have shown that GDNF plays an important role in the positive regulation of follicular growth and the development of preimplantation embryos in pigs and rats [7,9]. Evidence obtained from clinical data reveals that GDNF supplementation increases the number of metaphase II oocytes by promoting oocyte maturation and cumulus cell viability [10,11,12]. Given the important role of GDNF in the modulation of human follicular function, the regulation of GDNF in the human ovary has been a focus of research. However, it is unclear how GDNF is regulated and the underlying molecular mechanisms in the human ovary.

As one of the transforming growth factor-β (TGF-β) superfamily, TGF-β1 is a multifunctional cytokine that diversely modulates many cellular activities, including proliferation, differentiation and cell–cell interactions [13,14]. In reproductive biology, TGF-β1 is an important intrafollicular regulator that is involved in various steps of follicular development [15]. Targeted depletion of *Tgfb1* in male mice led to a significant reduction in sperm count and a decrease in serum levels of testosterone [16]. Targeted depletion of *Tgfb1* in female mice led to delayed sexual maturity, a reduction in the number of corpora lutea, embryos that were flushed from the oviduct or uterus and developmental failure of the preimplantation embryos [17]. Additionally, the serum concentrations of progesterone decreased by approximately 80% in *Tgfb1*^−/−^ mice compared with that of the wild-type mice [17]. We have previously shown that TGF-β1 is involved in a diverse range of essential roles during follicular development by regulating the transcriptomes of human granulosa cells and hormonal profiles in follicular fluid, including connexin 43, connective tissue growth factor, cyclooxygenase-2, matrix metalloproteinase-1, hyaluronan synthase 2, lysyl oxidase and steroidogenic acute regulatory protein [14,18,19,20,21,22,23]. Despite the important roles of GDNF and TGF-β1 in the regulation of follicular development, whether TGF-β is able to regulate the expression and production of GDNF in human granulosa cells remains to be determined.

Proprotein convertases (PCs) are a group of proteases of the subtilase family (nine mammalian PCs, including PCSK1-9) that proteolytically convert the precursor proteins to their active form [24]. Furin (also known as PCSK3 or paired basic amino acid cleaving enzyme, PACE) is the most well-known proprotein convertase and is a serine endoprotease that cleaves protein precursors at the carboxyterminal motif of basic residues, such as Arg–X–X–Arg and Lys/Arg–Arg [24,25]. The furin-like PCs process various precursor proteins in the secretory pathways, including growth factors, hormones, receptors, adhesion molecules, neuropeptides, glycoprotein and bacterial toxins [26]. As a distant member of the TGF-β superfamily, GDNF is a dimeric protein that is cleaved at a site (furin cleavage motif) between the latent associated peptide and the mature peptide [27,28]. In Chinese hamster ovary and human neural cells, PACE4 and several proprotein convertases, including furin, PCSK5 and PCSK7, cleave pro-GDNF into mature GDNF, of which furin is the main endoproteinase [29]. Our previous studies have shown that furin is expressed at a high level in human granulosa-lutein (hGL) cells and that furin is involved in the processing and synthesis of two intraovarian TGF-β superfamily members, activin A and TGF-β1 [30,31]. Notably, TGF-β1 has been shown to upregulate its own processing of enzyme furin in rat synoviocytes [32]. However, the functional role of furin in the processing and synthesis of GDNF in human granulosa cells has never been elucidated. Therefore, we sought to investigate the effect of TGF-β1 on the expression and production of GDNF and the molecular mechanisms underlying this effect.

## 2. Materials and Methods

### 2.1. Culture of Primary and Immortalized hGL Cells

Primary hGL cells were collected from patients undergoing oocyte retrieval during in vitro fertilization treatment. The Research Ethics Board of the University of British Columbia approved the study protocol and all patients signed a written informed consent form. The primary hGL cells were collected from pooled follicle fluid and isolated by the density centrifugation as previously described [33]. SVOG, the nontumorigenic immortalized human granulosa cell line, was previously produced by simian virus 40 large T-antigen transfection of human granulosa-luteal cells and was selected to explore the biological behavior of hGL cells because of the similarity between SVOG and primary hGL cells [34]. Cells were counted with a hemocytometer and cell viability was assessed with 0.04% trypan blue exclusion. The SVOG cells were seeded in six-well plates (2–4 × 10^5^ cells per well) and cultured in DMED/F12 (Sigma-Aldrich Corp.) medium supplemented with 10% charcoal/dextran-treated fetal bovine serum (HyClone Laboratories Inc., Logan, UT), 100 U/mL of penicillin, 100 μg/mL of streptomycin sulfate (Invitrogen, Life Technologies) and 1 × GlutaMAX (Invitrogen, Life Technologies). The medium was changed every other day in all the experiments. The cells were cultured in serum-free medium for 24 h before specific treatment.

### 2.2. Antibodies and Reagents

A polyclonal rabbit anti-furin convertase antibody (PA1-062; diluted at 1:1000; RRID: AB_2105077) was obtained from Thermo Fisher Scientific (Beverly, MA), and a monoclonal mouse anti-α-Tubulin antibody (sc-23948; diluted at 1:2000; RRID: AB_2493181) was obtained from Santa Cruz Biotechnology (Santa Cruz, CA, USA). A goat anti-human GDNF antibody (AF-212-NA; diluted at 1:1000; RRID: AB_2111398) was obtained from R&D Systems (Minneapolis, MN). Polyclonal rabbit anti-SMAD2 (#3103; diluted at 1:1000; RRID: AB_490816), anti-SMAD3 (#9523; diluted at 1:1000; RRID: AB_2193182), and anti-SMAD4 (#9515; diluted at 1:2000; RRID: AB_2193344) antibodies were obtained from Cell Signaling Technology (Beverly, MA). Mouse myeloma cell-derived recombinant human TGF-β1 was obtained from R&D Systems (Minneapolis, MN). Furin inhibitor II (SCP0148) and SB431542 were obtained from Sigma Aldrich.

### 2.3. Real-Time RT-qPCR

The cells were washed with cold PBS and total RNA was extracted with TRIzol reagent (Invitrogen) according to the manufacturer’s instructions. RNA (3 μg) was reverse-transcribed into first-strand cDNA with random primers and Moloney murine leukemia virus reverse transcriptase (Promega). RT-qPCR was performed on the Applied Biosystems 7300 real-time PCR system equipped with 96-well optical reaction plates. The primer sequences used for SYBR GREEN RT-qPCR were as follows: SMAD2, 5′-GCCTTTACA GCTTCTCTGAACAA-3′ (sense) and 5′ATGTGGCAATCCTTTTCG AT-3′ (antisense); SMAD3, 5′-CCC CAG CAC ATA ATAACT TGG-3′ (sense) and 5′-AGG AGA TGG AGC ACC AGAAG-3′ (antisense); SMAD4, 5′-TGG CCC AGG ATC AGT AGGT-3′ (sense) and 5′-CATCAACACCAATTCCAGCA-3′ (antisense); GDNF,5′-CGCTGAGCAGTGACTCAAATA-3′ (sense) and 5′-TGGAATTCTCTGGGTTGGCA-3′ (antisense); GAPDH,5′GAGTCAACGGATTTGGTCGT-3′ (sense) and 5′-GACAAGCTTCCCGTTCTCAG-3′(antisense); and FURIN, 5′-CCTTCTTCCGTGGGGTTAG-3′ (sense) and 5′-GCAGTTGCAGCTGTCATGTT-3′ (antisense). Each 20 μL RT-qPCR reaction contained 1× SYBR Green PCR master mix. (Applied Biosystems), 20 ng cDNA, and 250 nM of each specific primer. Alternatively, a TaqMan gene expression assay was used to detect ACVR1B (ALK4, Hs00244715_m1), TβRI (ALK5, Hs00610320_m1) and GAPDH (Hs02758991_g1) (Applied Biosystems) in triplicate on corresponding cDNA samples. For each 20-μL TaqMan reaction, 100 ng of cDNA was mixed with 10 μL of 2× TaqMan gene expression master mix (Applied Biosystems) and 1 μL of 20× TaqMan gene expression probe. The specificity of each assay was validated by a dissociation curve analysis and agarose gel electrophoresis of the PCR products. Three separate experiments were performed on different cultures and each sample was assayed in triplicate. A mean value was used to determine the mRNA levels using the computer Cq method with the formula 2^−ΔΔCq^. GAPDH was used as the reference gene.

### 2.4. Western Blot Analysis

After treatment, the cells were washed with cold PBS and lysed in lysis buffer (Cell Signaling Technology) containing a protease inhibitor cocktail (Sigma-Aldrich) for 30 min at 4 °C. The extracts were centrifuged at 12,000 *g* for 15 min at 4 °C to remove cellular debris. A DC protein assay (Bio-Rad Laboratories, Inc.) was used to determine protein concentration. Forty micrograms of protein from each sample were separated by 10% sodium dodecyl sulfate-polyacrylamide gel electrophoresis (SDS-PAGE) (Invitrogen, USA) and transferred onto polyvinylidene fluoride membranes for 1.5 h. After 1 h in blocking buffer containing 5% nonfat dry milk and 0.05% Tween, the membrane was incubated overnight at 4 °C with relevant primary antibodies. The membranes were washed three times with TBS-T for 1 h, incubated with peroxidase-conjugated secondary antibodies (Bio-Rad Laboratories Inc.) for 1 h and washed three times with TBS-T for 30 min. The protein bands were detected using enhanced chemiluminescence reagents or SuperSignal West Femto Chemiluminescence Substrate (Pierce), followed by exposure to CLXPosure film (Thermo Fisher). The membranes were stripped with stripping buffer at 50 °C for 30 min and reprobed with total SMAD2/3/4 or GAPDH antibodies as loading controls. Films were scanned and quantified by densitometry using Scion imaging software (Scion Corp).

### 2.5. Small Interfering RNA Transfection

We performed transient knockdown assays with an ON-TARGET plus SMART pool targeting control or a separate ON-TARGET plus SMART pool targeting ALK4, ALK5, SMAD2, SMAD3, SMAD4, furin or TGFBR2: ALK4 (L-004925-00-0005), ALK5 (L-003929-00-0005), SMAD2 (L-003561-00-0005), SMAD3 (L-020067-00-0005), SMAD4 (L-003902-00-0005), furin (L-005882-00-0005) or TGFBR2 (L-003930-00-0005) from Dharmacon (Lafayette, CO). Cells were precultured in antibiotic-free DMEM/F-12 medium containing 10% fetal serum until they reached 50–60% confluence and then transfected with 25 nM siRNA using Lipofectamine RNA iMAX (Life Technologies) for 24 h or 48 h, as previously described [35]. The knockdown efficiency for each target was analyzed using RT-qPCR or a Western blot analysis.

### 2.6. Measurement of Secreted GDNF

Following the specific treatment, the culture medium was collected and stored immediately at −80 ℃ until analysis. A human GDNF-specific ELISA kit was used in accordance with the manufacturer’s protocol (Thermo Fisher). Each sample was measured in triplicate and the level of secreted GDNF was normalized relative to the total cellular protein content.

### 2.7. Statistical Analysis

The results were analyzed by one-way ANOVA, followed by Tukey’s multiple comparison tests and are presented as the mean ± standard error of the mean of at least three independent experiments. *p* < 0.05 was considered statistically significant.

## 3. Results

### 3.1. TGF-β1 Induces GDNF Expression in Immortalized and Primary hGL Cells

**Figure 1:**
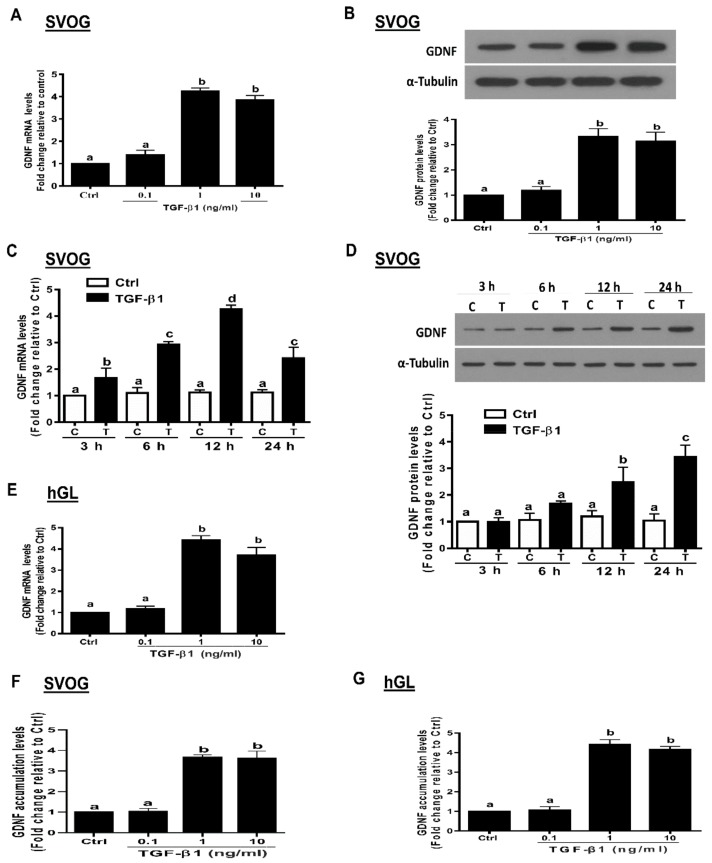
TGF-β1 upregulates the expression of GDNF in human granulosa-lutein cells. (**A**,**B**) SVOG cells were treated with the vehicle control (PBS) or various concentrations (0.1, 1, or 10 ng/mL) of TGF-β1 for 12 h (**A**) and 24 h (**B**), and the mRNA (**A**) and protein (**B**) levels of GDNF were examined using RT-PCR (**A**) and Western blot analysis (**B**), respectively. (**C**,**D**) SVOG cells were treated with 1 ng/mL TGF-β1 for 3, 6, 12 or 24 h, and the mRNA (**C**) and protein (**D**) levels of GDNF were examined using RT-PCR (**C**) and Western blot analysis (**D**), respectively. (**E**) Primary human granulosa-lutein (hGL) cells were treated with vehicle control or various concentrations (0.1, 1, or 10 ng/mL) of TGF-β1 for 12 h, and the mRNA levels of GDNF were examined using RT-qPCR. (**F**,**G**) SVOG (**F**) and hGL (**G**) cells were treated with vehicle control or various concentrations (0.1, 1, or 10 ng/mL) of TGF-β1 for 24 h, and the accumulated levels of GDNF in the conditioned medium were examined using an enzyme immunoassay. The results are expressed as the mean ± SE of at least three independent experiments. The values with different lower-case letters are significantly different (*p <* 0.05). If a pair of values is significantly different (*p* < 0.05), the values have different subscript letters (a vs. b or b vs. c) assigned to them. Ctrl, Control; C, Control; T, TGF-β1 treatment.

Because the range of the average concentrations of TGF-β1 in human follicular fluid (0.236–18.03 ng/mL) [36], we thus used the concentrations of 0.1–10 ng/mL TGF-β1 in the present study. Initially, we investigated the effect of recombinant human TGF-β1 (TGF-β1) on the expression of GDNF by treating SVOG cells with vehicle control (PBS) or varying concentrations (from 0.1, 1 to 10 ng/mL) of TGF-β1 for 12 h. As shown in Figure 1A, treatment with 1 and 10 ng/mL TGF-β1 significantly induced an increase in the mRNA levels of GDNF in SVOG cells. Similarly, the Western blot analysis showed that the same concentrations (1 and 10 ng/mL) of TGF-β1 induced a similar effect on the protein levels of GDNF after 24 h of treatment (Figure 1B). We then chose a concentration of 1 ng/mL TGF-β1 to perform the subsequent experiments. The timecourse studies showed that TGF-β1 (1 ng/mL) induced an increase in the mRNA levels of GDNF for different periods of time (3–24 h) (Figure 1C). Similarly, TGF-β1 (1 ng/mL) induced an increase in the protein levels of GDNF for different periods of time (12–24 h) (Figure 1D). Primary hGL cells were used to confirm the stimulatory effects of TGF-β1 on the expression of GDNF. Consistently with the results in SVOG cells, TGF-β1 (1 or 10 ng/mL for 12 h) significantly induced an increase in the mRNA levels of GDNF in primary hGL cells (Figure 1E). To investigate whether the TGF-β1-induced upregulation of GDNF expression contributes to an increase in GDNF production, we used an enzyme immunoassay to examine the accumulated levels of GDNF in the conditioned medium after incubation with TGF-β1. The results showed that TGF-β1 (1 or 10 ng/mL) induced an increase in the accumulated levels of GDNF in the conditioned medium of SVOG and primary hGL cells (Figure 1F,G).

### 3.2. TGF-β1 Induces Furin Expression in Immortalized and Primary hGL Cells

**Figure 2:**
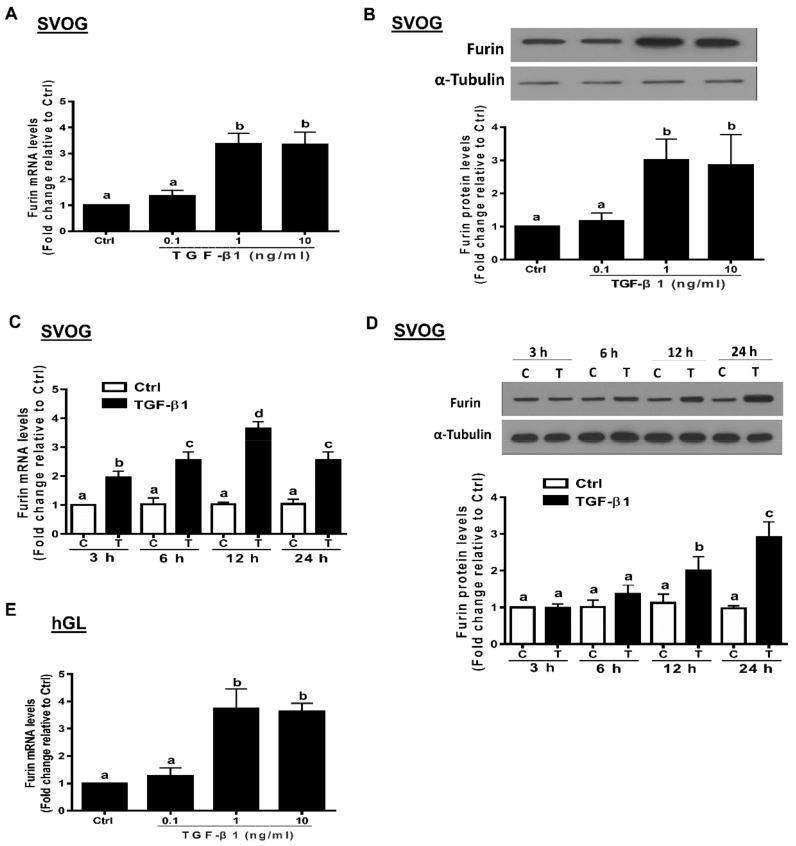
TGF-β1 upregulates the expression of furin in human granulosa-lutein cells. (**A**,**B**) SVOG cells were treated with vehicle control (PBS) or various concentrations (0.1, 1, or 10 ng/mL) of TGF-β1 for 12 h (**A**) and 24 h (**B**), and the mRNA (**A**) and protein (**B**) levels of furin were examined using RT-PCR (**A**) and Western blot (**B**), respectively. (**C**,**D**) SVOG cells were treated with 1 ng/mL TGF-β1 for 3, 6, 12 or 24 h, and the mRNA (**C**) and protein (**D**) levels of furin were examined using RT-PCR (**C**) and Western blot analysis (**D**), respectively. (**E**) hGL cells were treated with vehicle control or various concentrations (0.1, 1, or 10 ng/mL) of TGF-β1 for 12 h, and the mRNA levels of furin were examined using RT-PCR. The results are expressed as the mean ± SE of at least three independent experiments. Values with different lower-case letters are significantly different (*p <* 0.05). If a pair of values is significantly different (*p* < 0.05), the values have different subscript letters (a vs. b or b vs. c) assigned to them.

TGF-β1 has been shown to modulate the gene expression of the converting enzyme furin in various cells [32,37,38]. To investigate the effects of TGF-β1 on the expression of furin in hGL cells, we treated SVOG cells with varying concentrations of TGF-β1. The results show that TGF-β1 (1 or 10 ng/mL for 12 h) stimulation led to an increase in the mRNA levels of furin in SVOG and primary hGL cells (Figure 2A,E). Similarly, TGF-β1 (1 or 10 ng/mL for 24 h) stimulation led to an increase in the protein levels of furin in SVOG cells (Figure 2B). Additionally, TGF-β1 (1 ng/mL) stimulation for varying periods of time (starting at 3 h with the peak effect observed at 12 h) induced an increase in the mRNA levels (Figure 2C). Similarly, TGF-β1 (1 ng/mL) stimulation induced an increase in the protein levels, starting at 12 h, and the observed effect persisted until 24 h (Figure 2D).

### 3.3. The TGF-β Type II Receptor TβRII Mediates The TGF-β1-Induced Expression of GDNF and Furin in SVOG Cells

**Figure 3:**
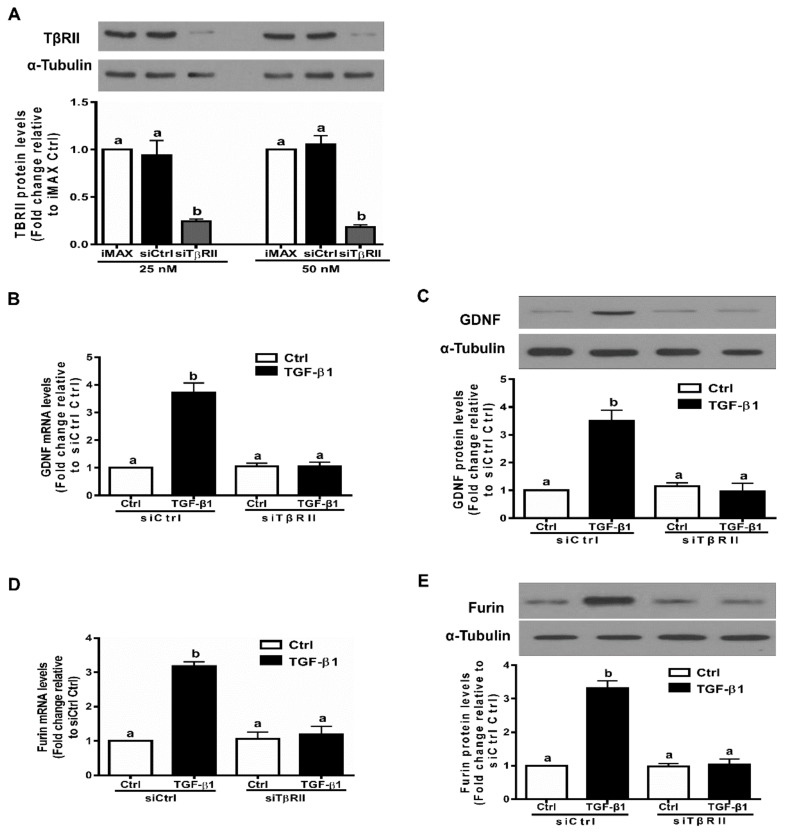
TβRII mediates the TGF-β1-induced upregulation of GDNF and furin in SVOG cells. (**A**) SVOG cells were transfected for 48 h with the transfection reagent (iMAX), control siRNA (siCtrl; 25 or 50 nM) or TβRII siRNA (siTβRII; 25 or 50 nM), and the protein levels of TβRII were examined using Western blot analysis. (**B**,**C**) SVOG cells were transfected for 48 h with 25 nM siCtrl or 25 nM siTβRII and then treated with vehicle control or 1 ng/mL TGF-β1 for an additional 12 h (**B**) of 24 h (**C**). The mRNA (**B**) and protein (**C**) levels of GDNF were examined using RT-qPCR (**B**) and Western blot analysis (**C**), respectively. (**D**,**E**) SVOG cells were transfected for 48 h with 25 nM siCtrl or 25 nM siTβRII and then treated with vehicle control or 1 ng/mL TGF-β1 for an additional 12 h (**D**) of 24 h (**E**). The mRNA (**D**) and protein (**E**) levels of furin were examined using RT-qPCR (**D**) and Western blot analysis (**E**), respectively. The results are expressed as the mean ± SE of at least three independent experiments. Values with different lower-case letters are significantly different (*p* < 0.05). If a pair of values is significantly different (*p* < 0.05), the values have different subscript letters (a vs. b or b vs. c) assigned to them. siCtrl, control siRNA.

In many cell types, ligands of the TGF-β superfamily exert their cellular activities by interacting with distinct sets of dual receptors, the type I and type II receptors (belonging to transmembrane Ser/Thr kinase receptors) [39]. Indeed, TβRII is thought to be the primary type II receptor through which TGF-β1 directly binds to and forms a ligand-receptor complex [40]. To confirm that TβRII is the type II receptor that mediates the TGF-β1-induced cellular activities in hGL cells, we used the siRNA-based inhibition approach. We first evaluated the knockdown efficiency of TβRII siRNA using Western blot analysis. As shown in Figure 3A, transfection with 25 nM or 50 nM siTβRII for 48 h induced a decrease in the protein levels of TβRII in SVOG cells. Notably, knockdown of TβRII completely abolished the TGF-β1-induced stimulatory effects on the mRNA and protein levels of GDNF in SVOG cells (Figure 3B,C). Similarly, knockdown of TβRII completely abolished the TGF-β1-induced stimulatory effects on the mRNA and protein levels of furin in SVOG cells (Figure 3D,E).

### 3.4. ALK5 is the Principal Type I Receptor that Mediates the Induced Expression of GDNF and Furin by TGF-β1 in SVOG Cells

**Figure 4:**
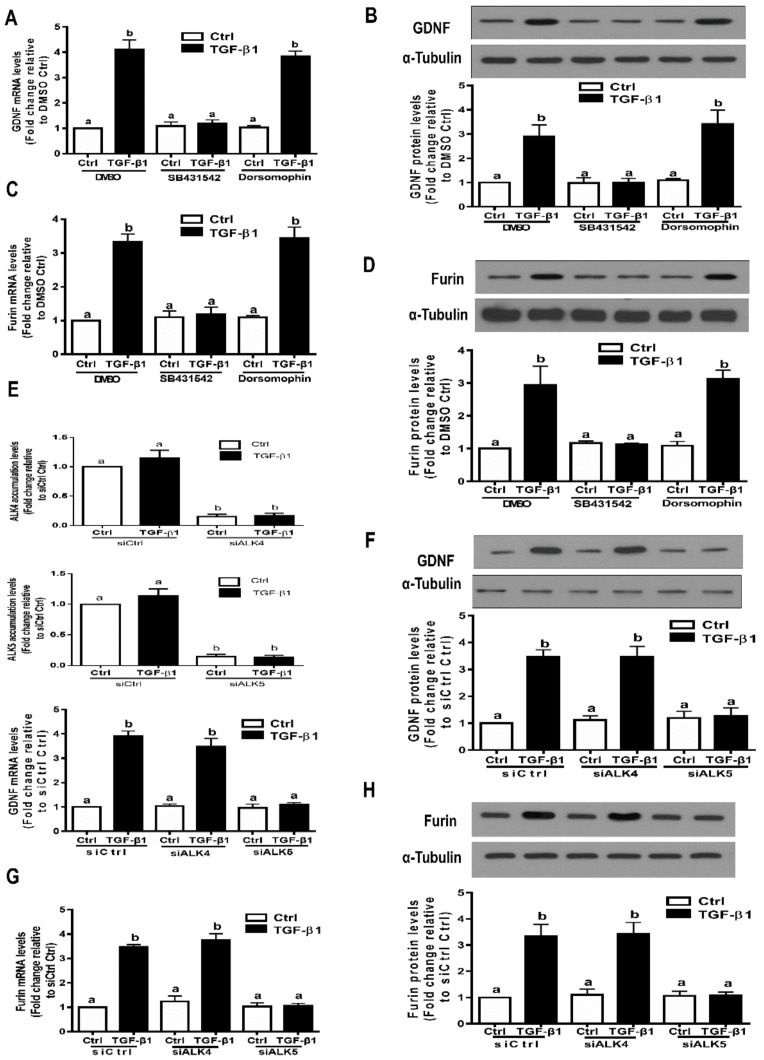
The TGF-β type I receptor ALK5 is required for the TGF-β1-induced upregulation of GDNF and furin in SVOG cells. (**A**,**B**) SVOG cells were pretreated with the vehicle control (dimethyl sulfoxide, DMSO), 10 µM SB431542 or 10 µM dorsomorphin dihydrochloride (dorsomorphin) for 1 h and then treated with 1 ng/mL TGF-β1 for an additional 12 h (**A**) or 24 h (**B**). The mRNA (**A**) and protein (**B**) levels of GDNF were examined using RT-qPCR (**A**) and Western blot analysis (**B**), respectively. (**C**,**D**) SVOG cells were pretreated with the vehicle control, 10 µM SB431542 or 10 µM dorsomorphin for 1 h and then treated with 1 ng/mL TGF-β1 for an additional 12 h (**C**) or 24 h (**D**). The mRNA (**C**) and protein (**D**) levels of furin were examined using RT-qPCR (**C**) and Western blot analysis (**D**), respectively. (**E**,**F**) SVOG cells were transfected for 48 h with 25 nM siCtrl, 25 nM si ALK4 or 25 nM siALK5 and then treated with 1 ng/mL TGF-β1 for an additional 12 h (**E**) or 24 h (**F**). The mRNA (**E**) and protein (**F**) levels of GDNF were examined using RT-qPCR (**E**) and Western blot analysis (**F**), respectively. (**G**,**H**) SVOG cells were transfected for 48 h with 25 nM siCtrl, 25 nM si ALK4 or 25 nM siALK5 and then treated with 1 ng/mL TGF-β1 for an additional 12 h (**G**) or 24 h (**H**). The mRNA (**G**) and protein (**H**) levels of furin were examined using RT-qPCR (**G**) and Western blot analysis (**H**), respectively. The results are expressed as the mean ± SE of at least three independent experiments. Values with different lower-case letters are significantly different (*p* < 0.05). If a pair of values is significantly different (*p* < 0.05), the values have different subscript letters (a vs. b or b vs. c) assigned to them.

Both ALK1 and ALK5 have been shown to be expressed in neural cells and mediate TGF-β1-induced downstream signaling pathway and cellular activities [41]. However, ALK1 is not expressed in human granulosa cells. We next investigated which TGF-β type I receptor (activin receptor-like kinase 1-7, also known as ALK1-7) is required for the TGF-β1-induced expression of GDNF and furin in hGL cells by using a dual inhibition approach, including small molecule kinase inhibitors and siRNA-based knockdown inhibition. Two established small molecular ATP-competitive inhibitors were used in the present study. SB431542 is an inhibitor that specifically binds to the ATP domain of ALK4, ALK5 and ALK7, and dorsomorphin is a potent inhibitor that specifically binds to the ATP domain of ALK2, ALK3 and ALK6 [42,43]. The results showed that pretreatment with SB431542 (10 µM) for 1 h prior to the addition of TGF-β1 (1 ng/mL) completely abolished the upregulation of GDNF mRNA and protein by TGF-β1 (Figure 4A,B). However, pretreatment with dorsomorphin (10 µM) for 1 h prior to the addition of TGF-β1 (1 ng/mL) did not influence the stimulatory effects of TGF-β1 on GDNF expression (Figure 4A,B). Similarly, pretreatment with SB431542, but not dorsomorphin, for 1 h prior to the addition of TGF-β1 (1 ng/mL) completely abolished the upregulation of furin mRNA and protein by TGF-β1 (Figure 4C,D). Given the possible off-target effects induced by these inhibitors, we used a siRNA-based knockdown approach to determine which ALK is the principal type I receptor that mediates the TGF-β1-induced effects in hGL cells. The knockdown efficiency of ALK4 and ALK5 was confirmed using real-time RT-qPCR (Figure 4E). The knockdown results showed that targeted depletion of ALK5 using siRNA transfection completely abolished the upregulation of GDNF mRNA and protein by TGF-β1 in the cells (Figure 4E,F). However, targeted depletion of ALK4 did not influence these stimulatory effects of TGF-β1 (Figure 4E,F). Similarly, targeted depletion of ALK5, but not ALK4, completely abolished the upregulation of furin mRNA and protein by TGF-β1 (Figure 4G,H).

### 3.5. The SMAD3-SMAD4 Signaling Pathway is Required for the Upregulation of GDNF and Furin by TGF-β1 in SVOG Cells

**Figure 5:**
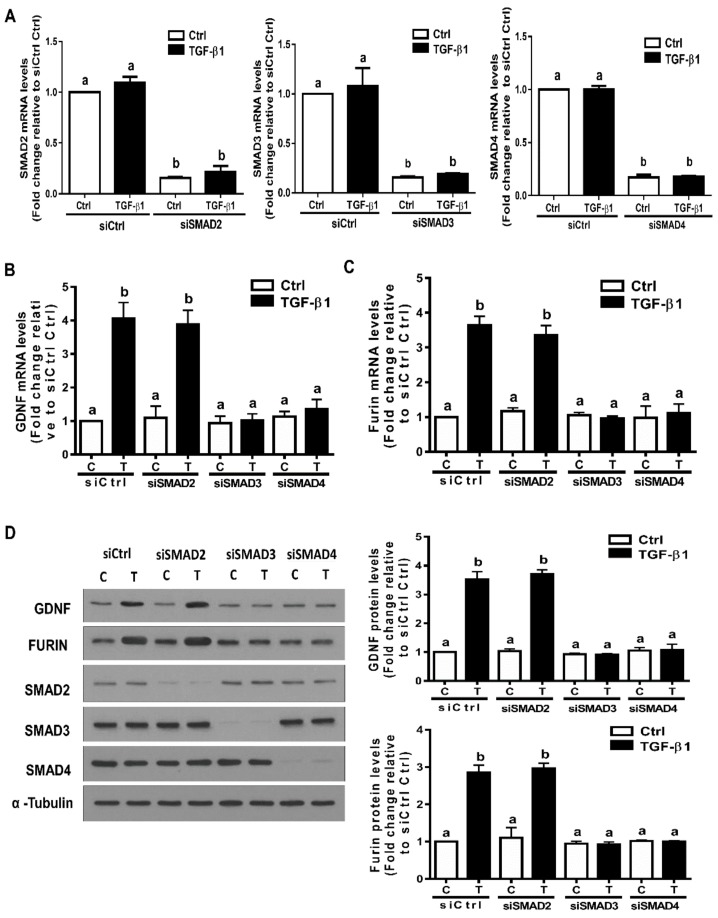
The SMAD3-SMAD4 signaling pathway is required for the TGF-β1-induced upregulation of GDNF and furin in SVOG cells. (**A**) SVOG cells were transfected with 25 nM siCtrl, 25 nM siSMAD2 25 nM siSMAD3 or 25 nM siSMAD4 for 48 h. The mRNA levels of SMAD2, SMAD3 and SMAD4 were examined using RT-qPCR. (**B**,**C**) SVOG cells were transfected for 48 h with 25 nM siCtrl, 25 nM siSMAD2, 25 nM siSMAD3 or 25 nM siSMAD4 and then treated with the vehicle control or 1 ng/mL TGF-β1 for an additional 12 h. The mRNA levels of GDNF (**B**) and furin (**C**) were examined using RT-qPCR. (**D**) SVOG cells were transfected for 48 h with 25 nM siCtrl, 25 nM siSMAD2, 25 nM siSMAD3 or 25 nM siSMAD4 and then treated with the vehicle control or 1 ng/mL TGF-β1 for an additional 24 h. The protein levels of GDNF, furin, SMAD2, SMAD3 and SMAD4 were examined using Western blot analysis. The results are expressed as the mean ± SE. Values with different lower-case letters are significantly different (*p* < 0.05). If a pair of values is significantly different (*p* < 0.05), the values have different subscript letters (a vs. b or b vs. c) assigned to them.

In the canonical signaling pathway, SMAD2, SMAD3 and SMAD4 are the principal downstream mediators of TGF-β1-induced cellular activities in hGL cells [18,20,44]. To investigate the involvement of these SMADs in the expression of GDNF and furin induced by TGF-β1, we transfected SVOG cells using siRNA-mediated knockdown of SMAD2, SMAD3 and SMAD4. RT-qPCR was used to evaluate the knockdown efficiency of each SMAD. Specifically, the results show that the mRNA levels of each SMAD significantly decreased after 48 h of siRNA-mediated knockdown (Figure 5A). Notably, knockdown of SMAD3 completely abolished the increased mRNA levels of GDNF and furin induced by TGF-β1 (Figure 5B,C). However, knockdown of SMAD2 did not have these effects (Figure 5B,C). Additionally, knockdown of SMAD4 completely blocked the stimulatory effects of TGF-β1 on the mRNA levels of GDNF and furin (Figure 5B,C). Similarly, the Western blot analysis results show that knockdown of SMAD3 or SMAD4, but not SMAD2, completely blocked the stimulatory effects of TGF-β1 on the protein levels of GDNF and furin (Figure 5D,E).

### 3.6. Furin Is Involved in the Increased GDNF Production by TGF-β1 in hGL Cells

**Figure 6:**
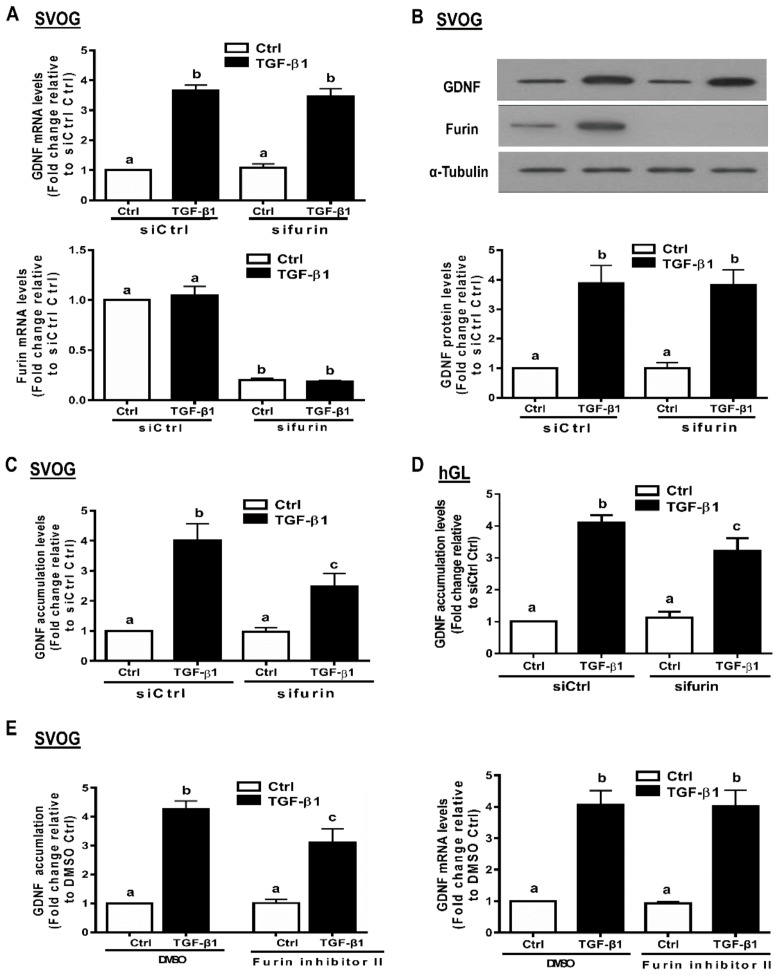
Furin is involved in the TGF-β1-induced increase in GDNF production in human granulosa-lutein cells. (**A**,**B**) SVOG cells were transfected for 48 h with 25 nM siCtrl or 25 nM siFurin and then treated with 1 ng/mL TGF-β1 for an additional 12 h (**A**) or 24 h (**B**). The mRNA (A) and protein (**B**) levels of GDNF and furin were examined using RT-qPCR (A) and Western blot analysis (B), respectively. (**C**,**D**) SVOG (**C**) or hGL (**D**) cells were transfected for 48 h with 25 nM siCtrl or 25 nM siFurin and then treated with 1 ng/mL TGF-β1 for an additional 24 h. The accumulated levels of GDNF in the conditioned medium were examined using an enzyme immunoassay. (**E**) SVOG cells were pretreated with DMSO or 10 µM furin inhibitor II for 1 h and then treated with 1 ng/mL TGF-β1 for an additional 24 h. The accumulated levels of GDNF in the conditioned medium and the mRNA levels of GDNF were examined using an enzyme immunoassay and RT-qPCR, respectively. The results are expressed as the mean ± SE. Values with different lower-case letters are significantly different (*p* < 0.05). If a pair of values is significantly different (*p* < 0.05), the values have different subscript letters (a vs. b or b vs. c) assigned to them.

Furin is the principal proprotein convertase for the proteolytic maturation of various proproteins, including GDNF [1]; however, it is unclear whether furin is involved in the production of GDNF in hGL cells. To investigate the functional role of furin in the increased GDNF production induced by TGF-β1 in hGL cells, siRNA-targeted downregulation of endogenous furin was used prior to TGF-β1 treatment. The results obtained from RT-qPCR and Western blot analysis revealed that knockdown of furin (transfection with 25 nM furin siRNA, siFurin for 48 h) decreased furin mRNA and protein by 80–90% (Figure 6A,B). Notably, knockdown of furin did not influence GDNF mRNA and protein in SVOG cells (Figure 6A,B). However, the enzyme immunoassay showed that knockdown of furin partially attenuated the increased GDNF accumulation in the conditioned medium induced by TGF-β1 in SVOG and primary hGL cells (Figure 6C,D). To further confirm the involvement of furin in the TGF-β1-induced increase in GDNF production, SVOG cells were pretreated with a specific furin inhibitor (furin inhibitor II) prior to TGF-β1 treatment. Consistently with the results obtained from the furin knockdown experiments, pretreatment with furin inhibitor II partially attenuated the stimulatory effects of TGF-β1 on GDNF accumulation without influencing the mRNA levels of GDNF (Figure 6E).

## 4. Discussion

The paracrine interactions between oocytes and the surrounding granulosa cells promote the growth and maturation of the oocytes [45,46,47]. GDNF is a critical granulosa cell-derived intraovarian factor that promotes the developmental potential of the oocyte in various species, including humans [9,10,11,12,48]. At present, the detailed molecular mechanisms involved in the production of GDNF in human granulosa cells remain largely unknown. In the present study, we provide the first data showing that TGF-β1 promotes the expression, maturation and secretion of GDNF in hGL cells. This function arises from the overall effect that TGF-β1 upregulates the expression of GDNF (at the transcriptional and translation levels) and that TGF-β1 upregulates the expression of proprotein convertase furin (the proteolytic processing enzyme for GDNF). The increased expression of GDNF by TGF-β1 is independent of furin because knocking down furin did not affect this effect. Notably, the TGF-β1-induced upregulation of furin seems to contribute to the enhanced production of mature GDNF because the knockdown of furin or the addition of a furin inhibitor partially attenuated this effect. The knockdown of furin or inhibition of the action of furin using a furin inhibitor did not completely abolish the TGF-β1-induced increase in GDNF production, indicating that other proprotein convertase may also be involved in the processing or secretion of GDNF. These findings indicate a novel autocrine/paracrine mechanism by which human granulosa cells can modulate their neighboring cells to promote the biosynthesis and secretion of mature GDNF required for oocyte maturation. Interestingly, we previously demonstrated a similar interactive regulatory effect showing that other TGF-β superfamily members, bone morphogenetic protein (BMP)4 and BMP7, enhanced activin A production by upregulating the expression of the inhibin βA subunit and furin in hGL cells [30]. These regulatory mechanisms provide a control loop of locally produced growth factors that may extend to other members of the TGF-β superfamily that are processed by proprotein convertases, including furin. Our most recent studies showed that oocyte- and granulosa cell-derived BMP6 increased the biosynthesis and maturation of TGF-β1 by upregulating the expression of furin in hGL cells [31]. Outside the reproductive system, TGF-β1 has been shown to upregulate the expression of furin. For instance, TGF-β1 increases the expression and activity of furin, which, in turn, enhances the TGF-β1-induced increase in matrix metalloproteinase 2 activity in cardiac fibroblasts [49]. Additionally, furin promotes the cleavage and activation of pro-TGF-β1 and pro-TGF-β2, and the formation of TGF-β2 further enhances the production of furin in glioma-initiating cells [50]. Experimental data obtained from in vitro studies revealed that the formation of mature TGF-β1 is markedly inhibited by the addition of a potent furin inhibitor [51]. Taken together, the previous studies and our studies suggest that the TGF-β1 increases the production of furin, the principle proprotein convertase that is primarily involved in the maturation and secretion of GDNF in hGL cells.

Information obtained from clinical studies has demonstrated that dysregulation or genetic variants in TGF-β-mediated signaling are associated with various reproductive disorders, including polycystic ovary syndrome, ovulation dysfunction and even ovarian cancers [52,53,54]. A comprehensive understanding of ligand-receptor interactions will be tremendously useful in developing therapeutic strategies for TGF-β1-related ovarian disorders. Similarly to other TGF-β superfamily members, TGF-β1 exerts its cellular activity by interacting with a couple of type I and type II receptors [39]. In the present study, we used a dual inhibition approach combining kinase receptor inhibitors and small interfering RNA-mediated knockdown to investigate ligand-receptor interactions. The results show that the addition of a selective ALK4/5/7 receptor inhibitor SB431542 completely blocked the stimulatory effects of TGF-β1 on the expression of GDNF and furin. Moreover, using siRNA-mediated gene silencing, we demonstrated that ALK5 is the primary type I receptor that mediates TGF-β1-induced cellular activity in hGL cells. Similarly, knockdown of TβRII using siRNAs targeting TβRII completely abolished the upregulation of GDNF and furin induced by TGF-β1, indicating that TβRII is the principal type II receptor that is required for the TGF-β1-induced biological response in hGL cells. These findings provide useful information regarding the ligand–receptor interaction of TGF-β signaling in hGL cells. Indeed, acting as a TGF-β signaling inhibitor, SB431542 has been used as a therapeutic agent in various animal studies, including bone reconstruction and regeneration [55,56].

The receptor-regulatory SMADs (SMAD2 and SMAD3) and common SMAD4 are canonical downstream mediators of TGF-β1 signaling in various mammalian cells. Our previous studies have shown that treatment with exogenous TGF-β1 induces the phosphorylation of SMAD2 and SMAD3 in hGL cells [18,44]. To investigate the functional roles of these canonical mediators in the increased expression of GDNF and furin by TGF-β1, we used siRNA-mediated gene silencing of the individual SMADs. The results show that knockdown of SMAD3 completely abolished the increased expression of GDNF and furin by TGF-β1, whereas knockdown of SMAD2 did not have any effect. In addition, knockdown of SMAD4 completely abolished the effect of TGF-β1 on the expression of GDNF and furin. These findings indicate that the SMAD3-SMAD4 signaling pathway is the downstream effector that mediates the increased expression of GDNF and furin by TGF-β1 in hGL cells. In human hepatic stellate cells, knockdown of SMAD2/3 abolishes the TGF-β-induced increase in GDNF [57]. In human liver cells (HepG2 cells), the expression of furin is induced and regulated by TGF-β1 via the MEK/p42/p44 MAPK and SMAD2-SMAD4 signaling pathways [38]. In human glioma-initiating cells, TGF-β2 controls Furin activity in an ALK5-dependent manner via the ERK/MAPK pathway [38]. Collectively, these studies suggest that the signaling pathway that mediates the TGF-β1-induced regulation of furin expression is cell-type specific. Our previous studies showed that BMP6 upregulates the expression of furin, an effect that is mediated by different type I receptors, ALK2 and ALK3, and different downstream signaling mediators, SMAD1/5/8-SMAD4, in hGL cells [31]. Therefore, BMP6 uses ALK2/ALK3-mediated SMAD1/5/8-SMAD4, but TGF-β1 uses SMAD3-SMAD4 as the intracellular signaling pathway to modulate the expression of furin in the same cell type.

However, in this study, we did not investigate the potential cellular activity and biological function of the secreted GDNF in hGL cells. Recent studies have shown that GDNF and its functional receptors are expressed at high levels in the human ovary and that GDNF signaling is involved in the direct control of various ovarian functions [8]. GDNF exerts its cellular activity through a ligand–receptor interaction. In many mammalian cells, two GDNF receptors, the GDNF family receptor-α1 (GFRα1) and RET receptor tyrosine kinase, are involved in this interaction [58]. Because the endogenous TGF-β is required for the exogenous GDNF-mediated neuroprotective effect in the peripheral nervous system, it has been hypothesized that the GDNF signaling pathway has crosstalk with the TGF-β signaling pathway [59]. Additionally, TGF-β1 enhances cell responsiveness to GDNF by recruiting the glycosyl-phosphatidylinositol-anchored GFRα1 to the plasma membrane in neural cells [60]. Whether TGF-β1 can recruit GFRα1 and enhance the cell responsiveness to GDNF in hGL cells remains to be elucidated. Based on our findings and previous studies, we hypothesize that TGF-β1 amplifies the biological effects of GDNF by inducing the expression of GDNF and recruiting GDNF receptors to the plasma membrane.

**Figure 7:**
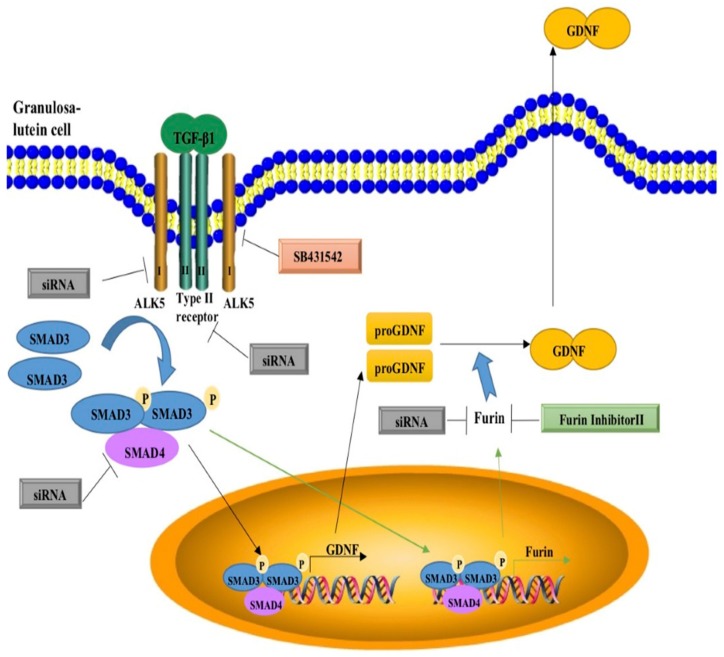
Proposed model for the upregulation of furin and GDNF production by TGF-β1 in human granulosa-lutein cells. TGF-β1 signals to granulosa cells by binding to TGF-β type II receptors (TβRII) and TGF-β type I receptors (ALK5). The activated type I receptors phosphorylate receptor-regulated SMAD3, which binds SMAD4 (common SMAD) to form a heterotrimeric complex that translocates to the nucleus to upregulate GDNF and furin expression. These effects can be abolished by the type I receptor inhibitor SB431542 or by siRNA targeting TβRII, ALK5, SMAD3 or SMAD4. Increased production of mature GDNF occurs via enhanced production as well as proteolytic processing of the proGDNF subunit, the latter occurring due to TGF-β1-induced production of its proprotein convertase, furin. Indeed, the TGF-β1-induced increase in GDNF production is partially reduced by pretreatment with furin siRNA or furin inhibitor II, indicating that furin is involved in the TGF-β1-induced increase in GDNF production and secretion.

In conclusion, this study demonstrates that TGF-β1 increases the biosynthesis and secretion of mature GDNF by inducing the expression of pro-GDNF in hGL cells. Additionally, the TGF-β1-induced increase in GDNF production is partially reduced by pretreatment with furin siRNA or furin inhibitor II, indicating that furin is involved in the TGF-β1-induced increase in GDNF production and secretion. Our study also shows that this cellular effect is most likely via a TβRII/ALK5-mediated SMAD3-SMAD4 signaling pathway (Figure 7). These findings provide a novel regulatory mechanism by which an intraovarian growth factor (TGF-β1) influences the production and secretion of another growth factor (GDNF) through a paracrine or autocrine interaction in hGL cells. Moreover, the related information deepens our understanding of broader roles for these intraovarian factors (TGF-β1, GDNF and furin) in the regulation of follicular function during the periovulatory phase.
